# Serum Asprosin Levels in Patients with Hidradenitis Suppurativa: A Case–Control Study

**DOI:** 10.3390/diagnostics16162621

**Published:** 2026-08-18

**Authors:** Murat Doğan, Elif Çetinkaya, Harbiye Dilek Canat, Ali Mert Gök, Burak Yıldız, Zafer Türkoğlu, İbrahim Halil Yavuz

**Affiliations:** 1 Department of Dermatology, Kaçkar State Hospital, 53300 Rize, Türkiye; 2 Department of Dermatology, Bakırköy Dr. Sadi Konuk Training and Research Hospital, 34147 İstanbul, Türkiye; 3 Department of Dermatology, Başakşehir Çam and Sakura City Hospital, 34480 İstanbul, Türkiye; 4 Department of Dermatology, Eyüpsultan State Hospital, 34050 İstanbul, Türkiye; 5 Department of Dermatology, Gaziosmanpaşa Training and Research Hospital, 34255 İstanbul, Türkiye

**Keywords:** hidradenitis suppurativa, metabolic syndrome, asprosin

## Abstract

**Background:** Hidradenitis suppurativa (HS) is a chronic inflammatory skin disease involving complex interactions between inflammatory and metabolic pathways. Asprosin is a glucogenic adipokine involved in glucose homeostasis, which has been investigated in relation to obesity, insulin resistance, and inflammatory processes. This study aimed to evaluate serum asprosin levels in patients with HS and to investigate their associations with disease severity and selected metabolic and inflammatory characteristics. **Methods:** This single-center, case–control study included 44 patients with HS and 44 age- and sex-matched healthy controls. Sociodemographic, clinical, anthropometric, metabolic, and inflammatory parameters were recorded. Serum asprosin levels were measured using an enzyme-linked immunosorbent assay, while disease severity was assessed using Hurley staging and the International Hidradenitis Suppurativa Severity Score System (IHS4). Multivariable regression analyses were performed using log-transformed asprosin concentrations to evaluate the independent association between HS status and serum asprosin after adjustment for relevant metabolic and lifestyle-related factors. **Results:** Serum asprosin levels were significantly lower in patients with HS than in healthy controls in the unadjusted analysis (30.07 ± 28.15 vs. 40.94 ± 33.11 ng/mL, respectively; *p* = 0.028). However, HS status was not independently associated with serum asprosin levels after adjustment for BMI, smoking status, metabolic syndrome, age, and sex or after adjustment for BMI, smoking status, fasting glucose, triglycerides, and HDL cholesterol. Additionally, BMI showed a consistent negative association with serum asprosin levels across all three models. Serum asprosin was not significantly associated with Hurley stage, IHS4 score, or metabolic syndrome, and ROC analysis demonstrated poor discriminatory performance of serum asprosin, with an area under the curve of 0.364, a sensitivity of 43.2%, and a specificity of 38.6%. **Conclusions:** Serum asprosin levels were lower in patients with HS than in healthy controls in the unadjusted analysis; however, this difference was not independently associated with HS after adjustment for relevant confounding factors. The absence of associations with disease severity and the poor discriminatory performance in ROC analysis do not support serum asprosin as a diagnostic or disease severity biomarker for HS. Thus, further prospective and mechanistic studies are required to clarify the biological significance of altered asprosin levels in HS.

## 1. Introduction

Hidradenitis suppurativa (HS) is a chronic inflammatory skin disease that primarily affects the axillary, inguinal, perianal, and inframammary regions—areas rich in apocrine glands—and is characterized by painful nodules, abscesses, sinus tracts, and scar formation [1]. Clinically, this disease progresses in phases of flare-ups and remissions, significantly impacting the individual’s quality of life. Although the etiology and pathogenesis of HS have not been fully elucidated, genetic predisposition, lifestyle factors, obesity, smoking, hormonal factors, and changes in the skin microbiome are believed to play significant roles [1,2]. The process, which begins with the obstruction of hair follicles, progresses through follicular hyperkeratosis, rupture, and inflammatory infiltration, leading to the development of sinus tracts and fistulas [3]. Histopathologically, a cellular infiltrate dominated by Th1, Th17, B cells, plasma cells, and neutrophils is observed, with the inflammation resulting from these immunological responses being responsible for disease chronicity and tissue destruction [4,5,6].

Comorbidities are quite common in patients with hidradenitis suppurativa. Additionally, systemic diseases such as obesity, diabetes, dyslipidemia, hypertension, metabolic syndrome (MetS), and polycystic ovary syndrome (PCOS) frequently coexist with HS and may exacerbate disease severity [7,8]. A strong association has been reported between the components of MetS and HS. In the literature, MetS prevalence in HS patients ranges from 10.4% to 50.6% [9], and it has been shown that its incidence is approximately 2.2 times higher than that in the general population [10]. Further, it has been reported that the prevalence of obesity is 3.5 times higher [11], the prevalence of diabetes mellitus (DM) is 2.8 times higher [12], and the risk of dyslipidemia is 1.7 to 2.5 times higher [11]. These findings suggest that there may be a bidirectional relationship between HS and metabolic disorders. Just as chronic inflammation triggers metabolic disorders, it is suggested that pro-inflammatory cytokines derived from adipose tissue may contribute to the pathogenesis of HS.

Asprosin is a recently identified glucogenic adipokine involved in glucose homeostasis and energy regulation [13]. Moreover, altered circulating asprosin levels have been reported in several metabolic conditions, including obesity, insulin resistance, and diabetes, although findings may vary across populations and clinical settings [14,15]. Experimental and clinical studies have also suggested potential interactions between asprosin and inflammatory processes [16]. However, the biological significance of altered asprosin levels in chronic inflammatory skin diseases remains unclear.

Given the frequent coexistence of metabolic abnormalities and chronic inflammation in patients with HS, investigating metabolic mediators such as asprosin may provide additional insight into the complex metabolic characteristics of this disease. Therefore, this study was conducted to evaluate serum asprosin levels in patients with HS and to investigate their potential associations with disease severity and selected metabolic and inflammatory parameters.

## 2. Materials and Methods

### 2.1. Study Design and Setting

This single-center, case–control study evaluated serum asprosin levels in HS patients and healthy controls. It also investigated the potential association between circulating asprosin concentrations and HS status, disease severity, and selected metabolic and inflammatory characteristics. Additionally, associations with common metabolic comorbidities, including obesity and metabolic syndrome, were also evaluated. Thus, this study was designed to explore the relationship between circulating asprosin levels and the clinical and metabolic phenotype of HS rather than to establish a pathogenetic or causal role for asprosin.

### 2.2. Study Population

This study included adults aged 18–65 years who presented to the Dermatology Outpatient Clinic of Başakşehir Çam ve Sakura City Hospital between October and December 2024 and were diagnosed with HS. The participants provided written informed consent and satisfied the inclusion criteria. The exclusion criteria comprised systemic autoimmune, inflammatory, infectious, metabolic, cardiovascular, hepatic, renal, or malignant diseases; pregnancy or lactation; active infection; neurological disorders; recent use of antibiotics or immunosuppressive/immunomodulatory agents; probiotic/prebiotic use; and adherence to restrictive or specific diets.

### 2.3. Data Collection for Secondary Objectives

After obtaining informed consent, demographic data, anthropometric measurements, and blood pressure were recorded for all participants, and disease-specific data, including onset age, disease duration, and treatment history, were additionally collected from HS patients. Disease severity was assessed using Hurley staging and IHS4, while quality of life was evaluated with the Dermatology Life Quality Index (DLQI). MetS was diagnosed according to the joint IDF/AHA/NHLBI criteria, and inflammatory markers (CRP and ESR) and systemic inflammatory indices (NLR, PLR, and SII) were calculated from CBC data.

### 2.4. Blood Sample Collection and Laboratory Techniques

Venous blood samples were obtained from all participants following a minimum 8 h fast to evaluate baseline laboratory parameters (CBC, glucose, lipid profile, CRP, and ESR) and serum asprosin levels. For asprosin measurement, blood was collected into CAT Serum Sep Clot Activator tubes (BD Vacutainer, Becton, Dickinson and Company, Franklin Lakes, NJ, USA), allowed to clot at room temperature for one hour, and centrifuged at 1000× *g* for 20 min. The isolated serum was then aliquoted into Eppendorf tubes and stored at −80 °C until analysis. Subsequently, serum asprosin concentrations were quantified using a commercial Human Asprosin ELISA Kit (E4095hu, BT Lab, Jiaxing, Zhejiang, China) in an authorized laboratory while strictly following the manufacturer’s instructions.

### 2.5. Ethical Approval

The study protocol was reviewed and approved by the Education Planning Committee and the Clinical Research Ethics Committee of Başakşehir Çam and Sakura City Hospital (Decision No: KAEK-11/11.09.2024.154, Date: 17 September 2024). All participants provided written informed consent prior to their inclusion in the study, and the entire investigation was conducted in strict accordance with the principles of the Declaration of Helsinki and Good Clinical Practice guidelines.

### 2.6. Sample Size

The control group consisted of 44 individuals who presented during the same time, had no systemic or dermatological conditions other than tinea unguium, and were matched with the patient group in terms of age and gender.

### 2.7. Statistics

Statistical analyses were performed using SPSS version 26.0 (IBM Corp., Armonk, NY, USA). Specifically, continuous variables were assessed for normality using the Kolmogorov–Smirnov test. Normally distributed variables were compared using the independent-samples *t*-test, whereas the Mann–Whitney U test was used for non-normally distributed variables. Comparisons among more than two groups were performed using one-way analysis of variance or the Kruskal–Wallis test, as appropriate, while categorical variables were compared using the chi-square test or Fisher’s exact test when appropriate. Correlations between continuous variables were assessed using Pearson or Spearman correlation coefficients according to data distribution.

To evaluate the independent association between HS status and serum asprosin levels, multivariable regression analyses were performed using log-transformed serum asprosin concentrations as the dependent variable. Three prespecified models were evaluated to assess whether HS status was independently associated with serum asprosin levels after adjustment for potential confounding factors. Model 1 was adjusted for BMI; Model 2 additionally included smoking status, metabolic syndrome, age, and sex. Lastly, Model 3 included BMI, smoking status, fasting glucose, triglycerides, and HDL cholesterol.

Receiver operating characteristic (ROC) curve analysis was performed to evaluate the discriminatory performance of serum asprosin and other laboratory parameters. Additionally, the area under the ROC curve (AUC), sensitivity, specificity, and optimal cutoff values were calculated. All statistical tests were two-sided, and a *p*-value < 0.05 was considered statistically significant.

## 3. Results

### 3.1. Demographic Characteristics

This study was conducted with a total of 88 participants, including 44 patients with HS and 44 healthy controls. In both groups, 43.2% (*n* = 19) of the participants were female, while 56.8% (*n* = 25) were male. Moreover, no statistically significant difference was found between the groups in terms of age or gender (*p* > 0.05).

In the patient group, body mass index (BMI: 31.70 ± 7.17 kg/m^2^), waist circumference (105.5 ± 1.86 cm), and weight were found to be significantly higher than in the control group (*p* < 0.001), with 56.4% of the HS patients (*n* = 24) classified as obese or morbidly obese. Additionally, the smoking rate in the HS group (79.5%) was found to be significantly higher than that in the control group (29.5%) (*p* < 0.001). Demographic characteristics are shown in Table 1.

### 3.2. Clinical Characteristics of HS Patients

The average age at disease onset was 25.7 ± 10.45 years, and the average time to diagnosis was 4.3 ± 5.52 years. A family history of HS was present in at least one family member in 79.5% of the participants. According to the Hurley staging system, 22.7%, 59.1%, and 18.2% of patients were classified as stages 1, 2, and 3, respectively, and according to the IHS4 scoring system, 29.5%, 52.3%, and 18.2% of patients had mild, moderate, and severe disease activity, respectively. The axilla (79.5%) and inguinal (65.9%) areas were identified as the most affected regions.

In the disease burden analysis, active pain was detected in 54.6% of patients (*n* = 24). In contrast, in the assessment using the Visual Analog Scale (VAS), 20.5% of patients reported moderate pain, while 9.1% reported severe pain. According to DLQI scores, more than half of the patients (54.5%) reported that their lives were negatively affected by the disease to a “large” or “extremely large” degree, while a statistically significant, positive, moderate correlation was found between the VAS pain score and the DLQI score (r = 0.588; *p* = 0.001) according to the correlation analysis.

It was determined that 65.9% of HS patients (*n* = 29) had previously undergone at least one surgical procedure due to HS, with analyses revealing a statistically significant association between surgical history and disease severity (Hurley stages) (*p* = 0.032). In particular, the fact that all stage 1 patients had a history of surgery indicates that this group consists of surgical candidates with chronic and recurrent nodules. Clinical characteristics are shown in Table 2.

### 3.3. Metabolic and Laboratory Characteristics

The prevalence of MetS in HS patients was found to be 50%, which is significantly higher than that in the control group (13.6%) (*p* = 0.001). In biochemical analyses, fasting glucose, systolic and diastolic blood pressure, and triglyceride (TG) levels were significantly higher in HS patients compared to the control group, while HDL levels were significantly lower (*p* < 0.05).

All values for CRP (4.75 mg/dL vs. 0.95 mg/dL), ESR (14 mm/h vs. 4 mm/h), NLR (1.93 vs. 1.6), and SII (559.5 vs. 445.3)—all of which are markers of systemic inflammation—were found to be statistically significantly higher in HS patients compared to healthy controls (*p* < 0.05).

A main finding of this study is that serum asprosin levels in the HS group (mean: 30.07 ng/mL) were significantly lower than those in the control group (mean: 40.94 ng/mL) (*p* = 0.028). Metabolic and laboratory characteristics are shown in Table 3.

When the relationship between asprosin levels and immune–inflammatory parameters was examined, a weak positive correlation (r = 0.302; *p* = 0.047) was observed between serum asprosin levels and lymphocyte count, while a weak negative correlation (r = −0.331; *p* = 0.028) was observed with the platelet-to-lymphocyte ratio (PLR). No significant association was found between asprosin levels and disease severity (Hurley, IHS4) or the presence of MetS (*p* > 0.05).

### 3.4. Serum Asprosin Levels

Another critical finding of this study is that serum asprosin levels were significantly lower in the HS group than in the healthy control group (30.07 ± 28.15 vs. 40.94 ± 33.11 ng/mL, respectively; *p* = 0.028).

When subgroup analyses and correlations of serum asprosin levels were examined, asprosin concentrations in obese HS patients were numerically higher than those in non-obese HS patients (39.4 ± 37.84 vs. 22.28 ng/mL, respectively), although this difference did not reach statistical significance (*p* = 0.128). Additionally, no significant association was found between serum asprosin levels and sex, smoking status, metabolic syndrome, Hurley stage, or IHS4 score (*p* > 0.05).

When the relationship between asprosin levels and immune–inflammatory parameters was examined, a weak positive correlation was observed between serum asprosin levels and lymphocyte count (r = 0.302; *p* = 0.047). A weak correlation was also observed between serum asprosin levels and the platelet-to-lymphocyte ratio (PLR) (r = 0.331; *p* = 0.028). Given the modest strength of these associations, they were considered exploratory. Moreover, no significant association was found between serum asprosin levels and disease severity or metabolic syndrome. Association between serum asprosin levels and clinical parameters are shown in Table 4.

### 3.5. Multivariable Analysis of Serum Asprosin Levels

Because serum asprosin levels showed a right-skewed distribution, multivariable regression analyses were performed using log-transformed serum asprosin concentrations as the dependent variable. Three prespecified models were evaluated to assess whether HS status was independently associated with serum asprosin levels after adjustment for potential confounding factors.

In Model 1, which adjusted for BMI, HS status was not independently associated with serum asprosin levels (B = −0.037, 95% CI: −0.357 to 0.283; *p* = 0.821). In contrast, BMI was independently and negatively associated with serum asprosin levels (B = −0.034, 95% CI: −0.056 to −0.012; *p* = 0.002).

In Model 2, which also adjusted for smoking status, metabolic syndrome, age, and sex, HS status remained not significantly associated with serum asprosin levels (B = −0.044, 95% CI: −0.423 to 0.335; *p* = 0.821). BMI remained independently and negatively associated with asprosin levels (B = −0.038, 95% CI: −0.068 to −0.009; *p* = 0.010), while smoking status (B = −0.079, 95% CI: −0.430 to 0.272; *p* = 0.660), metabolic syndrome (B = 0.215, 95% CI: −0.181 to 0.611; *p* = 0.287), age (B = −0.006, 95% CI: −0.020 to 0.007; *p* = 0.344), and sex (B = 0.113, 95% CI: −0.191 to 0.418; *p* = 0.466) were not significantly associated with serum asprosin levels.

In Model 3, which adjusted for BMI, smoking status, fasting glucose, triglycerides, and HDL cholesterol, HS status was again not independently associated with serum asprosin levels (B = 0.013, 95% CI: −0.363 to 0.390; *p* = 0.945). Additionally, BMI remained negatively associated with serum asprosin levels (B = −0.034, 95% CI: −0.056 to −0.011; *p* = 0.003), whereas fasting glucose (B = −0.0003, 95% CI: −0.0031 to 0.0026; *p* = 0.848), triglycerides (B = −0.0003, 95% CI: −0.0015 to 0.0009; *p* = 0.607), HDL cholesterol (B = −0.004, 95% CI: −0.018 to 0.009; *p* = 0.545), and smoking status (B = −0.137, 95% CI: −0.492 to 0.217; *p* = 0.448) were not significantly associated with asprosin levels.

Overall, across all three prespecified multivariable model specifications, HS status was not independently associated with serum asprosin levels. The consistent negative association between BMI and serum asprosin levels suggests that differences in BMI may contribute to the lower asprosin concentrations observed in the unadjusted comparison between patients with HS and healthy controls, with the results of the multivariable regression models presented in Table 5.

### 3.6. ROC Analysis

**Figure 1:**
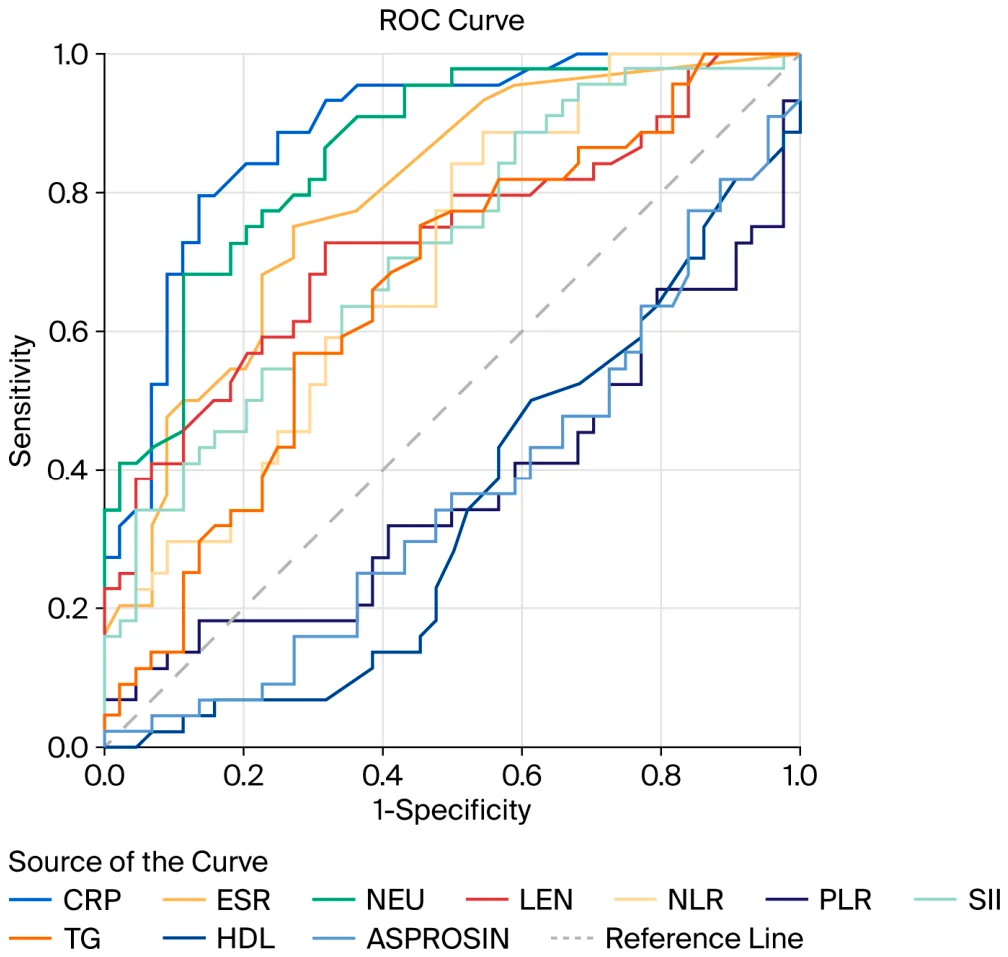
Comparison of the discriminatory performance of serum asprosin and inflammatory indices using ROC curves. CRP: C-reactive protein; ESR: erythrocyte sedimentation rate; NEU: neutrophil; LEN: lymphocyte; NLR: neutrophil-to-lymphocyte ratio; PLR: platelet-to-lymphocyte ratio; SII: systemic immune–inflammation index; TG: triglyceride; HDL: high-density lipoprotein. The reference line represents an AUC of 0.50.

In the ROC analysis evaluating discriminatory performance, CRP (AUC: 0.887) and neutrophils (AUC: 0.861) demonstrated the highest discriminatory performance. The AUC for serum asprosin was 0.364 (*p* = 0.028), indicating poor discriminatory performance despite statistical significance. At a cutoff value of 22.38 ng/mL, the sensitivity and specificity were 43.2% and 38.6%, respectively. These findings do not support serum asprosin as a clinically useful diagnostic biomarker for HS. ROC analysis is shown in Figure 1.

## 4. Discussion

Hidradenitis suppurativa (HS) is a chronic inflammatory skin disease characterized by follicular occlusion, rupture, and subsequent inflammatory tissue destruction. Increasing evidence indicates that keratinocytes play an active role in HS pathogenesis and contribute to follicular hyperkeratosis, inflammation, and sinus tract formation [1,2,3,5]. In addition to its cutaneous manifestations, HS is frequently associated with obesity, smoking, metabolic syndrome (MetS), and other cardiometabolic comorbidities [9,10,11,12,17,18,19]. These associations have increased interest in the interaction between inflammatory and metabolic pathways in HS. In this context, this study was conducted to investigate serum asprosin, a fasting-induced glucogenic protein hormone and adipokine involved in metabolic regulation, in patients with HS [15,20,21].

The main finding of this study is that serum asprosin levels were significantly lower in HS patients than in age- and sex-matched healthy controls in the unadjusted analysis. This finding is of interest because asprosin has been described as a metabolic hormone involved in fasting-induced glucose production and energy homeostasis [14]. Previous studies have reported associations between circulating asprosin levels and obesity, insulin resistance, diabetes, and other metabolic disorders, although the direction and strength of these associations may vary according to the studied population and metabolic context [15,16,20]. However, the observed difference in the present study should be interpreted cautiously because the HS and control groups were not matched for BMI, smoking status, waist circumference, or MetS, which may act as potential confounding factors when circulating adipokine concentrations are compared between groups.

To further investigate whether the difference in serum asprosin levels was independently associated with HS, multivariable regression analyses were performed using log-transformed asprosin concentrations. After adjustment for BMI, HS status was not independently associated with serum asprosin levels. This lack of association persisted after additional adjustment for smoking status, metabolic syndrome, age, and sex, as well as in a separate model including fasting glucose, triglycerides, and HDL cholesterol. In contrast, BMI showed a consistent negative association with serum asprosin levels across all three models. These findings suggest that the lower asprosin concentrations observed in the unadjusted comparison between HS patients and controls may be influenced, at least in part, by differences in BMI rather than representing an independent HS-specific alteration. Importantly, these results also emphasize that the observed between-group difference should not be interpreted as evidence of a direct or causal relationship between HS and circulating asprosin levels.

Our cohort also demonstrated a substantial cardiometabolic burden among patients with HS. BMI, waist circumference, fasting glucose, triglycerides, blood pressure, and MetS were higher in the HS group, whereas HDL levels were lower. These findings are consistent with previous studies demonstrating an increased prevalence of metabolic syndrome and cardiometabolic risk factors among patients with HS [9,10,11,12,13,18]. The high prevalence of MetS in our cohort further supports the importance of cardiometabolic risk assessment in HS patients. However, serum asprosin was not significantly associated with MetS or fasting glucose in our study. Thus, although metabolic abnormalities were more prevalent in the HS group, the relationship between these abnormalities and circulating asprosin was not straightforward.

An additional important finding was the absence of a significant association between serum asprosin levels and HS disease severity. Neither Hurley stage nor IHS4 score was significantly correlated with asprosin concentrations. Thus, the observed difference in asprosin levels between HS patients and controls did not translate into a relationship with the clinical severity of HS. This distinction is important because a difference in circulating levels between patients and controls does not necessarily indicate that a molecule reflects disease activity or severity. Accordingly, our findings do not support asprosin as a disease severity marker in HS. Given the relatively small sample size, particularly within individual Hurley stages and metabolic subgroups, these findings should nevertheless be confirmed in larger cohorts.

The biological explanation for the lower asprosin levels observed in the unadjusted comparison remains uncertain. Several studies have reported altered circulating asprosin concentrations in different metabolic and inflammatory conditions [15,16,20,22]. For example, reduced serum asprosin levels have been reported in obese children [23] and in patients with fibromyalgia [23]. However, these findings originate from different clinical populations and cannot be directly extrapolated to adults with HS. Similarly, lower circulating asprosin levels have been reported in patients with acromegaly, with possible relationships to altered glucose metabolism [22]. Although this observation is of interest, it cannot be directly applied to our cohort because acromegaly represents a distinct endocrine disorder and because we did not observe a significant association between asprosin and fasting glucose or MetS. Furthermore, fasting insulin, HOMA-IR, and OGTT were not assessed in the present study. Therefore, insulin resistance or altered glucose metabolism cannot be established as an explanation for the observed asprosin levels.

Inflammatory mechanisms may also be relevant to asprosin biology in HS, as HS is characterized by complex immune dysregulation involving multiple inflammatory pathways and cytokines [2,3]. Additionally, asprosin has been investigated in relation to metabolic and inflammatory processes [15,16,20]. Because inflammatory cytokines and oxidative stress markers were not directly measured, any potential relationship between inflammatory or oxidative pathways and altered asprosin levels should be regarded as hypothesis-generating rather than as a mechanism demonstrated by the present study [7].

The weak positive correlation observed between asprosin and lymphocyte count and the weak correlation observed between asprosin and PLR may provide preliminary clues regarding a possible relationship between asprosin and immune–inflammatory processes. Nevertheless, these correlations were modest and should be interpreted cautiously. Given the limited sample size and the absence of direct mechanistic measurements, these findings should be considered exploratory and should not be interpreted as evidence of a causal relationship between asprosin and immune dysregulation in HS.

Smoking was substantially more prevalent among HS patients than controls, consistent with the established association between smoking and HS [1,17,24]. Although smoking status was not significantly associated with serum asprosin levels in our cohort, its marked imbalance between groups represents a potential confounder; importantly, adjustment for smoking did not reveal an independent association between HS status and asprosin levels.

The discriminatory performance of serum asprosin was also assessed by ROC analysis. The AUC was 0.364, with a sensitivity of 43.2% and a specificity of 38.6%. These findings indicate poor discriminatory performance and do not support the use of serum asprosin as a diagnostic biomarker for HS. Therefore, although an unadjusted difference in serum asprosin concentrations was observed between patients and controls, this difference did not demonstrate clinically useful diagnostic discrimination.

In summary, this study demonstrated lower serum asprosin levels in patients with HS than in healthy controls in the unadjusted comparison. However, this difference was no longer statistically significant after adjusting for BMI and other relevant metabolic and lifestyle characteristics. Furthermore, asprosin was not associated with Hurley stage, IHS4 score, MetS, or most metabolic and inflammatory parameters, and ROC analysis demonstrated poor discriminatory performance. Therefore, the present findings do not support asprosin as a diagnostic or disease severity biomarker for HS. Rather, they provide preliminary and hypothesis-generating evidence that altered asprosin levels may be observed in the metabolic and inflammatory context of HS. Further prospective and mechanistic studies incorporating insulin resistance measures, body fat distribution, inflammatory cytokines, and oxidative stress markers are required to clarify the biological significance of asprosin in HS.

### Limitations

Several limitations of this study should be acknowledged. First, the single-center case–control design and relatively small sample size limit the generalizability of the findings and preclude conclusions regarding causality. Second, although the groups were matched for age and sex, they were not matched for BMI, smoking status, waist circumference, or MetS. Although multivariable analyses were performed to account for several of these potential confounders, residual confounding cannot be excluded. Third, the absence of fasting insulin, HOMA-IR, and OGTT measurements limited our ability to evaluate insulin resistance and glucose–asprosin interactions in detail. Fourth, detailed assessment of body fat distribution was not performed. Fifth, direct measurements of inflammatory cytokines and oxidative stress markers were not available, limiting mechanistic interpretation. Finally, the relatively small number of participants in individual Hurley stages and metabolic subgroups limited the statistical power of subgroup analyses. In addition, the relatively small sample size limited the number of covariates that could be reliably included in multivariable models.

## 5. Conclusions

In this case–control study, serum asprosin levels were significantly lower in patients with hidradenitis suppurativa than in age- and sex-matched healthy controls in the unadjusted analysis. However, this difference was no longer statistically significant after adjustment for BMI and other relevant metabolic and lifestyle characteristics. Furthermore, serum asprosin was not associated with Hurley stage, IHS4 score, metabolic syndrome, or most metabolic and inflammatory parameters, and ROC analysis demonstrated poor discriminatory performance.

These findings do not support serum asprosin as an independent diagnostic or disease severity biomarker for hidradenitis suppurativa. The lower asprosin levels observed in the unadjusted analysis may be influenced by differences in metabolic and lifestyle characteristics between HS patients and controls instead of representing an independent disease-specific alteration. Thus, further prospective and mechanistic studies are needed to determine whether altered asprosin levels have biological significance in HS.

## Figures and Tables

**Table 1:** Baseline demographic characteristics of HS patients and healthy controls.

Variable	HS Group (*n* = 44)	Healthy Group (*n* = 44)	*p*-Value
Age	32.61 ± 11.40	32.34 ± 9.74	0.789
Gender (female)	43.2% (*n* = 19)	43.2% (*n* = 19)	1.000
BMI (kg/m^2^)	31.70 ± 7.17	24.17 ± 3.85	<0.001
Smoking	79.5% (*n* = 35)	29.5% (*n* = 13)	<0.001

Data are presented as mean ± standard deviation for continuous variables and as percentages (*n*) for categorical variables. BMI: body mass index; HS: hidradenitis suppurativa. A *p*-value < 0.05 was considered statistically significant.

**Table 2:** Clinical characteristics and disease severity of the patients.

Variable	*n*	%
Family History	35	79.5%
Pain	24	54.6%
VAS		
No pain	24	54.5%
Mild pain	7	15.9%
Moderate pain	9	20.5%
Severe pain	4	9.1%
Hurley Stage		
Stage 1	10	22.7%
Stage 2	26	59.1%
Stage 3	8	18.2%
IHS4		
Mild	13	29.5%
Moderate	23	52.3%
Severe	8	18.2%
DLQI		
No effect at all on the patient’s life	5	11.4%
Small effect on the patient’s life	9	20.5%
Moderate effect on the patient’s life	6	13.6%
Very large effect on the patient’s life	21	47.7%
Extremely large effect on the patient’s life	3	6.8%
Surgery	29	65.9%
Lesion Site		
Axilla	35	79.5%
Inguinal	29	65.9%
Perineal	4	9.1%
Gluteal	6	13.6%
Intergluteal	1	2.3%
Inframammarian	5	11.4%
Intermammarian	10	22.7%
Lower abdomen	4	9.1%
Pubis	5	11.4%
Back of neck	3	6.8%

Data are presented as percentages (*n*). VAS: Visual Analog Scale; IHS4: International Hidradenitis Suppurativa Severity Score System; DLQI: Dermatology Life Quality Index. Multiple lesion sites may be present in a single patient.

**Table 3:** Metabolic and biochemical characteristics of patients and controls.

Variable	HS Group (*n* = 44)	Healthy Group (*n* = 44)	*p*-Value
Metabolic Syndrome	50% (22)	13.6% (6)	0.001
Fasting Glucose	102.73 ± 42.66	88.23 ± 15.73	0.025
Systolic Blood Pressure	123.02 ± 12.35	111.86 ± 11.23	0.001
Diastolic Blood Pressure	80.11 ± 7.32	72.8 ± 8.69	0.001
Triglycerides	146.27 ± 118.66	102.18 ± 65.68	0.010
HDL	42.14 ± 10.30	49.57 ± 13.05	0.004
CRP	11.5 ± 16.97	1.68 ± 2.45	0.001
ESR	16.45 ± 12.9	6.45 ± 7.04	0.001
NLR	2.14 ± 0.74	1.67 ± 0.46	0.002
SII	687.4 ± 405.8	457.01 ± 159.49	0.001
Asprosin	30.073 ± 28.15	40.94 ± 33.11	0.028

Data are presented as the mean ± standard deviation for continuous variables and as *n* (%) for categorical variables. HDL: high-density lipoprotein; CRP: C-reactive protein; ESR: erythrocyte sedimentation rate; NLR: neutrophil-to-lymphocyte ratio; SII: systemic immune–inflammation index. A *p*-value < 0.05 was considered statistically significant.

**Table 4:** Association between serum asprosin levels and clinical parameters.

Variable	Asprosin Mean ± SD	*p*-Value
Gender		0.627
Female	28.62 ± 22.15	
Male	31.97 ± 35.1	
Smoking	26.45 ± 15.16	0.932
Metabolic Syndrome	27.68 ± 31.74	0.716
Hurley Stage		0.342
Stage 1	30.31 ± 28.17	
Stage 2	29.7 ± 31.8	
Stage 3	30.99 ± 14.9	
IHS4		0.645
Mild	32.03 ± 41.6	
Moderate	28.1 ± 21.8	
Severe	32.5 ± 19.5	
Obesity	39.4 ± 37.84	0.128

SD: standard deviation; IHS4: International Hidradenitis Suppurativa Severity Score System.

**Table 5:** Multivariable regression analysis of serum asprosin levels.

Variable	Model 1 B (95% CI)	*p*	Model 2 B (95% CI)	*p*	Model 3 B (95% CI)	*p*
HS status	−0.037 (−0.357 to 0.283)	0.821	−0.044 (−0.423 to 0.335)	0.821	0.013 (−0.363 to 0.390)	0.945
BMI	−0.034 (−0.056 to −0.012)	0.002	−0.038 (−0.068 to −0.009)	0.010	−0.034 (−0.056 to −0.011)	0.003
Smoking status	—	—	−0.079 (−0.430 to 0.272)	0.660	−0.137 (−0.492 to 0.217)	0.448
Metabolic syndrome	—	—	0.215 (−0.181 to 0.611)	0.287	—	—
Age	—	—	−0.006 (−0.020 to 0.007)	0.344	—	—
Sex	—	—	0.113 (−0.191 to 0.418)	0.466	—	—
Fasting glucose	—	—	—	—	−0.0003 (−0.0031 to 0.0026)	0.848
Triglycerides	—	—	—	—	−0.0003 (−0.0015 to 0.0009)	0.607
HDL cholesterol	—	—	—	—	−0.004 (−0.018 to 0.009)	0.545

Model fit: Model 1, R^2^ = 0.141 (adjusted R^2^ = 0.120); Model 2, R^2^ = 0.175 (adjusted R^2^ = 0.114); Model 3, R^2^ = 0.152 (adjusted R^2^ = 0.090). Notes: The dependent variable was log-transformed serum asprosin concentration. B, regression coefficient; CI, confidence interval; BMI, body mass index; HS, hidradenitis suppurativa; HDL, high-density lipoprotein. Model 1 was adjusted for BMI; Model 2 was adjusted for BMI, smoking status, metabolic syndrome, age, and sex; Model 3 was adjusted for BMI, smoking status, fasting glucose, triglycerides, and HDL cholesterol.

## Data Availability

The data that support the findings of this study are available from the corresponding author upon reasonable request.

## Abbreviations

The following abbreviations are used in this manuscript:

HS	Hidradenitis suppurativa
BMI	Body mass index
IHS4	International Hidradenitis Suppurativa Severity Score System
